# The PVR-TIGIT/CD96/CD226 axis: a novel immune checkpoint target in ovarian cancer

**DOI:** 10.3389/fimmu.2026.1903882

**Published:** 2026-08-20

**Authors:** Mingyao Huang, Jiahui Zhang, Huiyan Huang, Li Liu, Yun Zou

**Affiliations:** 1 School of Basic Medicine Science, Key Laboratory of Translational Tumor Medicine in Fujian Province, Putian University, Putian, Fujian, China; 2 China Medical University, Shenyang, Liaoning, China; 3 Department of Nursing, The Fourth Hospital of Xianyou, Putian, Fujian, China; 4 Department of Anesthesiology, The Fourth Affiliated Hospital of China Medical University, Shenyang, Liaoning, China; 5 Department of Nephrology, The Fourth Affiliated Hospital of China Medical University, Shenyang, Liaoning, China

**Keywords:** CD155, immune checkpoint, immune regulation, immunotherapy, ovarian cancer

## Abstract

The poliovirus receptor (CD155) is an emerging immune checkpoint molecule involved in the suppression of anti-tumor immunity. In ovarian cancer, CD155 is frequently overexpressed and interacts with receptors such as TIGIT, CD96, and CD226 to modulate the activity of T cells, NK cells, and dendritic cells. These interactions contribute to immune cell exhaustion, regulatory T cell expansion, and impaired cytotoxic responses, facilitating tumor immune evasion. Recent studies have revealed that CD155 expression is influenced by tumor metabolism, autophagy, and cytokine signaling. Therapeutic strategies targeting the CD155 axis—through monoclonal antibodies, bispecific constructs, or engineered T/NK cells—are being actively explored and show promise in enhancing anti-tumor responses, particularly when combined with existing checkpoint inhibitors. This review provides a comprehensive overview of the structure, function, and receptor interactions of CD155 (PVR), with a particular focus on its immunoregulatory role in the ovarian tumor microenvironment. We discuss the signaling mechanisms of the TIGIT/CD96/CD226 axis, its impact on diverse immune cell populations, and the current landscape of therapeutic strategies targeting this pathway. Finally, we evaluate the potential of CD155 and its receptors as predictive biomarkers and therapeutic targets to overcome immune resistance and improve outcomes in ovarian cancer.

## 1 Introduction

Ovarian cancer remains one of the most lethal gynecological malignancies, primarily due to late-stage diagnosis and the high rate of recurrence after initial treatment (1, 2). Despite advancements in surgical techniques, chemotherapy, and targeted therapies, overall survival rates for patients with advanced ovarian cancer have seen only modest improvements (3). A key contributor to this poor prognosis is the ability of ovarian tumors to evade immune surveillance, enabling sustained growth and metastasis (4).

Recent studies have emphasized the critical role of the immune microenvironment in ovarian cancer progression. Within the tumor microenvironment (TME), the function of immune effector cells such as T cells and natural killer (NK) cells is often impaired, allowing cancer cells to escape immune-mediated destruction (5, 6). This immune escape is facilitated, in part, by the upregulation of immune checkpoint pathways, which suppress antitumor immunity and create an immunosuppressive niche favorable to tumor survival (7, 8).

Poliovirus Receptor (PVR), also known as cluster of differentiation 155 (CD155), a transmembrane glycoprotein involved in immune modulation, has emerged as a key player in immune evasion mechanisms in ovarian cancer (9, 10). CD155 interacts with various immune receptors, including TIGIT, CD96, and CD226, influencing the balance between immune activation and suppression. Through its preferential binding to inhibitory receptors such as TIGIT and CD96, CD155 promotes immune suppression by dampening T cell and NK cell activity, thereby facilitating tumor immune escape (11).

Given its central role in shaping the immunosuppressive microenvironment, CD155 has become a promising target for novel immunotherapeutic strategies in ovarian cancer. Targeting CD155, either alone or in combination with other immune checkpoint inhibitors, holds the potential to restore antitumor immunity and improve therapeutic outcomes (12). This review focuses on the role of CD155 in immune regulation within ovarian cancer, its contribution to tumor immune evasion, and the development of emerging therapeutic approaches aimed at disrupting this pathway.

## 2 Overview of the structure and function of CD155 and its receptor interactions

CD155, also referred to as Necl-5 or PVR, is part of the Nectin (nectin1-4) and Nectin-like (Necl1-5) families. It belongs to the Ca2+-independent immunoglobulin superfamily (IgSF) of cell adhesion molecules and was initially recognized as a poliovirus receptor (13). The CD155 gene is located on human chromosome 19, specifically in the q13.31–13.32 region, and encodes a precursor mRNA with eight exons. Alternative splicing of this mRNA produces four distinct protein isoforms: CD155α, CD155β, CD155γ, and CD155δ (14, 15). Of these, CD155α and CD155δ are transmembrane forms, while CD155β and CD155γ are soluble variants (soluble CD155, sCD155) (16). The transmembrane forms contain three immunoglobulin-like extracellular domains (V-C2-C2), a membrane-spanning region, and an intracellular cytoplasmic region. The α and δ isoforms differ only in the intracellular domain, with CD155α featuring an Immunoreceptor Tyrosine-based Inhibitory Motif (ITIM) (17). Soluble CD155 variants lack the transmembrane region encoded by exon 6 and can be secreted into the bloodstream and cerebrospinal fluid without requiring proteolytic cleavage (15, 16).

The majority of current research on CD155 centers on its transmembrane form, particularly CD155α, which is the largest variant and contains an ITIM domain (16). CD155 is expressed in both dendritic and tumor cells (18), and its levels are also increased in tumor-associated myeloid-derived suppressor cells (MDSCs) (19). Importantly, CD155 has been found to be expressed on malignant epithelial cells in ovarian cancer and shows a negative correlation with tumor-infiltrating lymphocytes. This suggests that CD155 may serve as a novel immunotherapeutic target in ovarian cancer (20).

**Figure 1:**
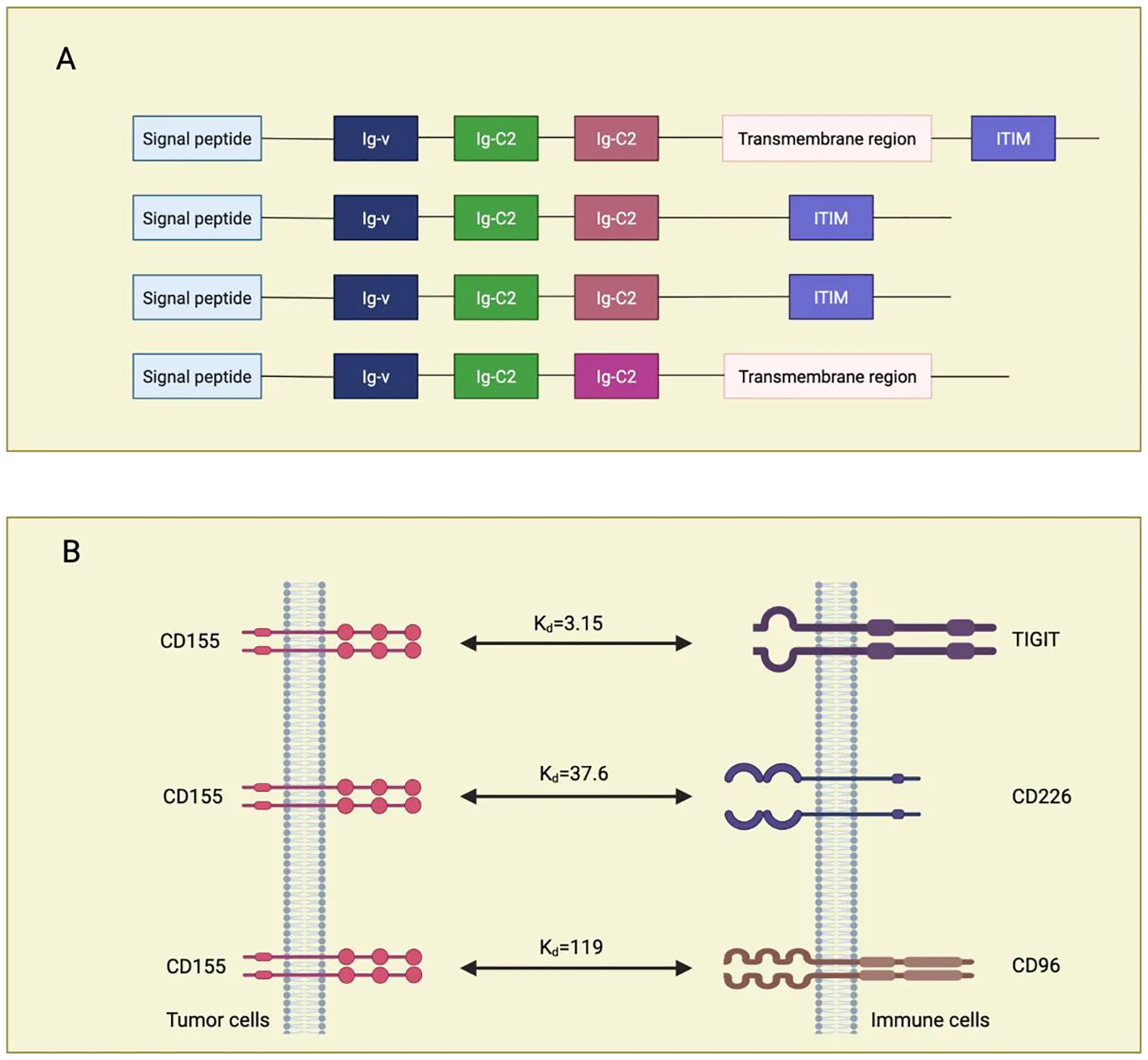
Overview of the structure and function of CD155 and its receptor interactions. **(A)** CD155 is located on the q13.31–q13.32 region of human chromosome 19 and gives rise to four isoforms—CD155α, CD155β, CD155γ, and CD155δ—through alternative splicing, with each isoform differing at the C-terminal due to variations in stop codon usage. CD155α and CD155δ are transmembrane forms containing an intracellular region, while CD155β and CD155γ are soluble variants (soluble CD155, sCD155). **(B)** CD155 is frequently overexpressed in tumor cells and interacts with immune cell surface receptors, including the inhibitory receptors TIGIT and CD96, as well as the activating receptor CD226. IgV, immunoglobulin variable domain; IgC, immunoglobulin constant domain; TM, transmembrane domain; ITIM, immunoreceptor tyrosine-based inhibitory motif; ITT, immunoglobulin tail tyrosine; YXXM, tyrosine-X-X-methionine motif. These interactions occur with varying binding affinities (Kd values in the nanomolar range: TIGIT-CD155 ~1 nM, CD226-CD155 ~5 nM, CD96-CD155 ~10 nM) and play a crucial role in modulating antitumor immune responses.

CD155 interacts with the receptors TIGIT, CD96, and CD226, each with distinct binding affinities, thereby regulating immune responses (**Figure 1**). Among these, CD155 exhibits the highest affinity for TIGIT, positioning TIGIT as a dominant inhibitory pathway in the CD155 axis (18). TIGIT, also known as Washington University Cell Adhesion Molecule (WUCAM) or V-set and Immunoglobulin Domain-Containing Protein 9 (VSIG9), was identified in 2009 as an immune checkpoint receptor (18). This type I transmembrane protein contains both an extracellular immunoglobulin variable (IgV) domain and an intracellular ITIM, with its inhibitory function dependent on the activation of both the ITIM and ITT motifs (21). TIGIT is expressed on various T cell subsets, including CD4+ T cells, CD8+ T cells, follicular helper T cells, and NK cells (21). Unlike the inhibitory function of TIGIT, CD226 (also known as DNAX Accessory Molecule-1, DNAM-1) primarily facilitates immune activation. As a member of the immunoglobulin superfamily (IgSF), CD226 contains two IgV domains arranged in parallel (22) and possesses an ITT or ITT-like signaling motif (23). It is expressed on T cells, NK cells, and monocytes (24), and is recognized as a marker of highly functional CD8+ cells, closely tied to NK cell-mediated immune surveillance (25). Adding further complexity, CD96, another IgSF member, comprises three extracellular Ig-like domains and an intracellular ITIM, along with a YXXM motif in humans (26–29). While CD96 generally promotes immunosuppressive effects upon binding to CD155, it may function as a co-stimulatory receptor under specific conditions (29, 30). It is primarily expressed on CD4+, CD8+ T cells, and NK cells (30) (**Figure 1**).

## 3 CD155-receptor interaction mediates immune response in ovarian cancer

### 3.1 CD155-TIGIT

TIGIT, also known as WUCAM, Vstm3, and VSIG9, is a member of the PVR/nectin family within the immunoglobulin superfamily. Its structure consists of an IgV domain, a type I transmembrane domain, and an intracellular region containing both an ITT motif and a conventional ITIM. By interacting with CD155, TIGIT suppresses immune cell signaling, thereby facilitating tumor immune evasion (31, 32). In humans, TIGIT is predominantly expressed on CD8+ T cells, NK cells, regulatory T cells (Tregs), and follicular helper T cells, with relatively low expression in naïve T lymphocytes (33).

**Figure 2:**
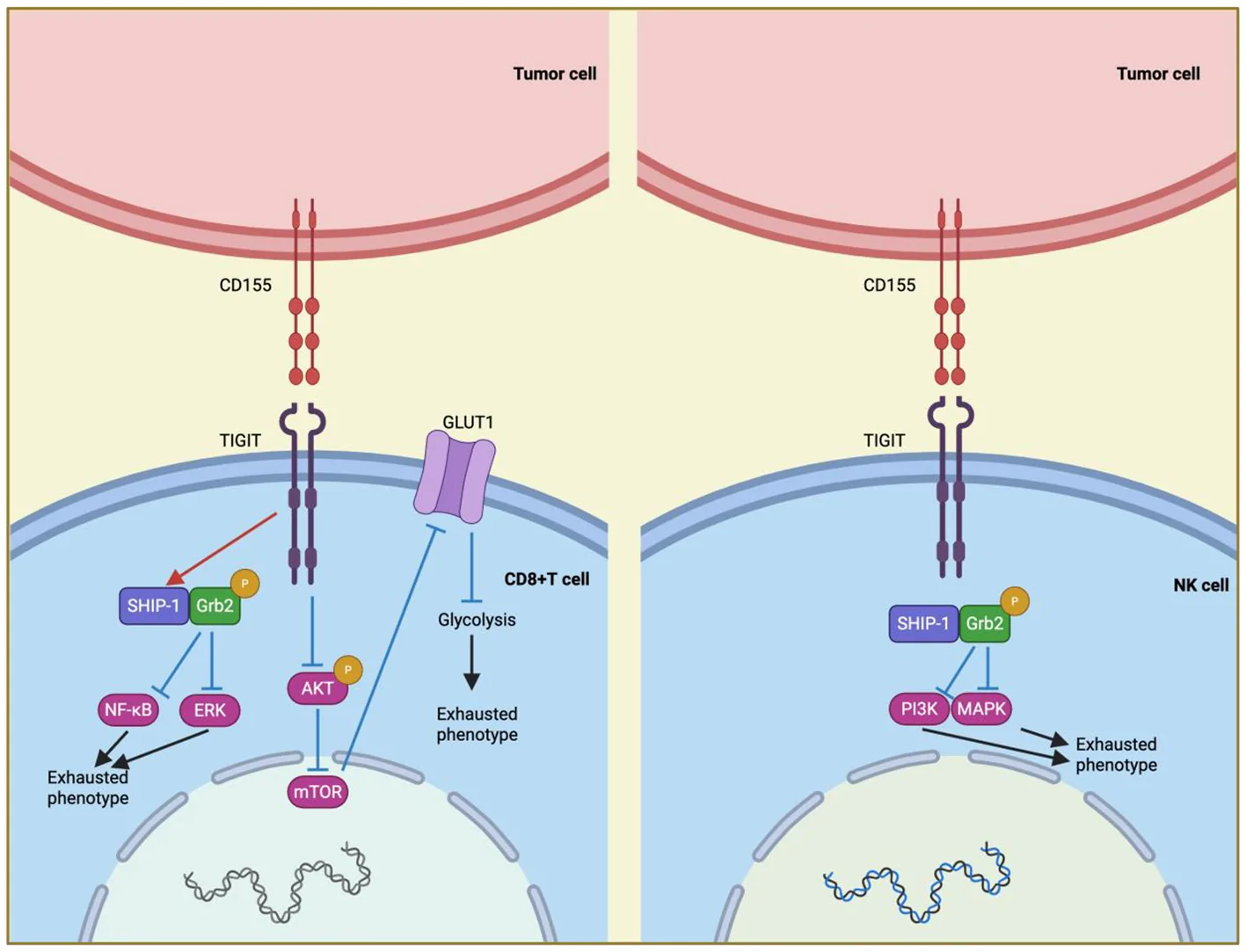
Interaction of CD155 with TIGIT in CD8^+^T and NK cells. CD155 expressed on tumor cells or antigen-presenting cells engages TIGIT on CD8^+^ T cells and NK cells. Upon binding, TIGIT’s ITT-like motif undergoes phosphorylation at Tyr225, enabling recruitment of the adaptor Grb2 and the phosphatase SHIP1. This signaling cascade inhibits PI3K and MAPK pathways, leading to reduced glycolysis, impaired cytokine production (IFN-γ, TNF-α), and diminished cytotoxic function. In NK cells, SHIP1 further inhibits TRAF6 auto-ubiquitination, blocking NF-κB activation and suppressing IFN-γ production. SHIP1, SH2-containing inositol phosphatase 1; Grb2, growth factor receptor-bound protein 2; TRAF6, TNF receptor-associated factor 6; PI3K, phosphoinositide 3-kinase; MAPK, mitogen-activated protein kinase; NF-κB, nuclear factor kappa-light-chain-enhancer of activated B cells; TCR, T cell receptor; IFN-γ, interferon-gamma; TNF-α, tumor necrosis factor-alpha.

The expression level of TIGIT in tumor-infiltrating CD8+ T cells is strongly correlated with tumor progression. Overexpression of TIGIT is a defining feature of the “exhausted” phenotype, which is characterized by reduced proliferation, impaired cytokine secretion, and diminished cytotoxic activity. This functional impairment weakens the anti-tumor immune response, allowing tumor cells to evade immune surveillance (34, 35). Notably, deep single-cell RNA sequencing (scRNA-seq) analysis of tumor samples from seven untreated patients with early or advanced high-grade serous ovarian cancer (HGSOC), along with five age-matched non-malignant ovarian samples, revealed that TIGIT is highly expressed on exhausted CD8+ T cells. Furthermore, *in vivo* studies demonstrated that TIGIT blockade significantly reduces tumor growth in mouse models of ovarian cancer (36). Critically, while these findings establish a correlative and causal link between TIGIT expression and T cell dysfunction in ovarian cancer, the small sample size (n=7) of the scRNA-seq study limits the generalizability of the conclusions, and whether this TIGIT^+^ exhausted signature is uniformly present across all HGSOC subtypes or varies with tumor stage, mutational burden, or prior treatment history remains unexplored. Moreover, the preclinical efficacy of TIGIT blockade in mouse models has not yet been consistently replicated in human ovarian cancer clinical trials, highlighting a critical gap between preclinical promise and clinical translation. Mechanistically, upon binding to its ligand CD155 and undergoing phosphorylation, TIGIT recruits SH2-containing inositol phosphatase 1 (SHIP-1), which in turn inhibits the activation of the NF-κB and ERK signaling pathways. This inhibition leads to the suppression of cytokine production by CD8+T cells, thereby dampening their anti-tumor activity (37). However, the relative contribution of this SHIP-1-dependent pathway versus other TIGIT-mediated inhibitory mechanisms, such as direct competition with CD226 for CD155 binding or TIGIT-induced trans-endocytosis of CD155, has not been systematically dissected in ovarian cancer models, and it remains unclear which mechanism predominates in the human TME. Moreover, evidence suggests that in the TME, TIGIT-induced CD8^+^ T cell exhaustion may be driven by the regulation of glucose metabolism (38, 39). In TIGIT^+^CD8^+^T cells, decreased AKT/mTOR phosphorylation leads to the downregulation of key metabolic genes, including GLUT1, HK1, HK2, and c-Myc, thereby impairing glycolysis and anabolic processes. This metabolic disruption contributes to T cell dysfunction, weakening their effector function. Importantly, both glucose supplementation and TIGIT blockade have been shown to restore GLUT1 expression, reverse T cell exhaustion, and enhance IFN-γ production, highlighting a potential avenue for improving anti-tumor immunity (40, 41). Nevertheless, the translational relevance of these metabolic findings warrants cautious interpretation. The glucose concentrations used in these *in vitro* studies may not faithfully recapitulate the complex and heterogeneous metabolic landscape of the ovarian tumor microenvironment, where nutrient availability, hypoxia, and lactic acid accumulation vary spatially and temporally. Furthermore, whether metabolic modulation, such as glucose supplementation, can be safely and effectively combined with TIGIT blockade *in vivo* without inadvertently promoting tumor growth or systemic metabolic disturbances has yet to be demonstrated. Additionally, a recent study identified elevated levels of CCL23 in the ascitic fluid of HGSOC patients, with plasma concentrations of CCL23 also significantly higher in HGSOC patients compared to healthy individuals. Further *in vitro* investigations revealed that macrophage-derived CCL23 induces TIGIT upregulation on CD8+T cells through the phosphorylation of GSK3β, a key regulator of the Wnt signaling pathway (42). This finding suggests a potential mechanism by which tumor-associated macrophages contribute to CD8+T cell exhaustion in the TME. Moreover, it establishes a novel link between Wnt signaling and CD8+T cell dysfunction, providing new insights into how Wnt/GSK3β activation may drive T cell exhaustion and facilitate immune evasion in HGSOC (**Figure 2**).

TIGIT induced “exhausted” phenotypes were also observed in NK cells. NK cells derived from ascites of patients with advanced ovarian cancer exhibit upregulated expression of the inhibitory receptor TIGIT. Notably, TIGIT blockade enhances the degranulation and IFN-γ production of CD56^dim^ NK cells from healthy donors when interacting with ovarian cancer cells, particularly in the presence of DNAM-1/CD155 engagement (43). Critically, this study primarily utilized *in vitro* co-culture systems employing healthy donor-derived NK cells rather than autologous tumor-infiltrating NK cells from patients. Healthy donor NK cells and patient ascites-derived NK cells differ substantially in phenotype, metabolic status, and functional reserve, raising the question of whether these findings accurately reflect the *in vivo* effects of TIGIT blockade in ovarian cancer patients. Moreover, the observed enhancement was dependent on concomitant DNAM-1/CD155 engagement, suggesting that the efficacy of TIGIT blockade may rely on the integrity of the CD226/CD155 axis, which is frequently downregulated on NK cells in advanced ovarian cancer, potentially constituting a resistance mechanism to this therapeutic strategy. Consistently, a study investigating the effects of artesunate on endometrial cancer found that artesunate treatment effectively suppressed TIGIT expression in NK cells, thereby enhancing their cytotoxicity. Mechanistically, artesunate regulated TIGIT expression through the induction of ATG5-dependent autophagy, highlighting a potential pathway by which autophagy modulation influences NK cell function within the TME (44). However, it is important to note that this study was conducted in endometrial cancer, which differs substantially from ovarian cancer in terms of immune microenvironment characteristics, including mutational burden, immune cell infiltration density, and cytokine profiles. Whether the artesunate-autophagy-TIGIT regulatory axis operates similarly in ovarian cancer remains unknown. Furthermore, this study primarily employed the NK92 cell line rather than primary patient-derived NK cells, and cell lines may exhibit important differences from primary NK cells in autophagic flux, metabolic demands, and receptor expression profiles, limiting direct extrapolation of these mechanistic insights to ovarian cancer patients. In addition, a recent study collecting peripheral blood and tumor biopsies from cervical cancer patients used flow cytometry to evaluate co-stimulatory receptor expression on exhausted NK cells. The results revealed a marked upregulation of TIGIT on peripheral exhausted NK cells, underscoring its potential role in NK cell dysfunction and immune evasion in cervical cancer (45). Nevertheless, this study only evaluated peripheral blood NK cells, whereas peripheral and tumor-infiltrating NK cells exhibit significant phenotypic and functional differences. Tumor-infiltrating NK cells typically display more pronounced exhaustion features and altered receptor expression. It therefore remains questionable whether peripheral TIGIT upregulation accurately reflects the functional status of NK cells at the tumor site. Additionally, this study was correlational in nature and did not establish a direct causal relationship between TIGIT upregulation and impaired NK cell function. The study also did not validate whether TIGIT blockade could restore NK cell anti-tumor function in cervical cancer. Likewise, Leung et al. demonstrated that TIGIT blockade markedly enhances NK cell-mediated cytotoxicity in ovarian cancer (46), reinforcing the therapeutic potential of targeting TIGIT to restore NK cell antitumor function specifically in ovarian cancer. While this study provides encouraging proof-of-concept evidence, it primarily relied on *in vitro* cytotoxicity assays and lacked *in vivo* animal model data to confirm the therapeutic efficacy of TIGIT blockade in enhancing NK cell function against ovarian cancer. More importantly, the study did not examine the durability of NK cell functional restoration following TIGIT blockade, nor its interplay with other immunosuppressive cells in the tumor microenvironment, such as regulatory T cells or myeloid-derived suppressor cells. These factors could substantially influence actual *in vivo* therapeutic outcomes. Interestingly, Yang et al. identified an autophagy-related gene, FOXO1, in ovarian cancer, which can induce autophagy in ovarian cancer cells and promote tumor metastasis in ovarian cancer cell lines. In addition, FOXO1 expression was found to be associated with the upregulation of TIGIT in tumor tissues (47). This finding is consistent with observations in endometrial cancer (44), suggesting that autophagy may regulate TIGIT expression on immune cells in gynecological malignancies. However, further studies are urgently needed to determine whether FOXO1 expression modulates TIGIT expression on tumor-infiltrating immune cells in ovarian cancer, to elucidate the underlying molecular mechanisms, and to identify the specific immune cell subsets involved. Then how does the CD155-TIGIT axis activate signal transduction in NK cells? Notably, upon binding to CD155, TIGIT’s ITT-like motif undergoes phosphorylation at Tyr225, enabling its interaction with the cytoplasmic adaptor Grb2, which subsequently recruits SHIP1. This recruitment inhibits PI3K and MAPK signaling, leading to diminished NK cell function (48). Furthermore, the binding of TIGIT to CD155 in NK cells triggers the phosphorylation of Tyr225 within TIGIT’s ITIM domain, facilitating its interaction with the newly identified adaptor β-arrestin 2, which subsequently recruits SHIP1. SHIP1 then inhibits the auto-ubiquitination of TNF receptor-associated factor 6 (TRAF6), thereby blocking NF-κB activation and ultimately suppressing IFN-γ production (49) (**Figure 2**).

Tregs play a crucial role in immune suppression by expressing high-affinity IL-2 receptors and secreting immunosuppressive cytokines. In human Tregs, several inhibitory receptors, such as TIGIT, PD-1, and CTLA-4, are abundantly expressed (50). Studies have shown that in tumor-bearing mice, TIGIT expression in Tregs is significantly elevated compared to normal mice. Notably, these TIGIT^+^ Tregs exhibit enhanced suppressive capacity, effectively inhibiting both the function and proliferation of activated CD8+T cells (51). Similarly, in a mouse model of ovarian cancer, TIGIT expression was markedly upregulated in immune cells, particularly in CD4^+^ Tregs. Importantly, anti-TIGIT treatment significantly impaired the immunosuppressive function of CD4+Tregs, suggesting that TIGIT expression reinforces their immunosuppressive activity (52). However, these findings are derived exclusively from murine models, and whether TIGIT^+^Tregs exert comparable immunosuppressive effects in human ovarian cancer patients remains largely unconfirmed, particularly with respect to clinical outcomes such as survival or immunotherapy response. Building on this, recent studies have identified a critical link between TIGIT+Tregs and focal adhesion kinase (FAK) signaling in the TME. Active FAK, characterized by tyrosine-576 phosphorylation within its catalytic domain, is significantly upregulated in advanced HGSOC, where it contributes to the maintenance of an immunosuppressive landscape. Notably, in an ovarian cancer mouse model, dual inhibition of FAK and TIGIT signaling through a combination of a FAK inhibitor and the TIGIT-blocking antibody (1B4) led to a substantial increase in tumor-infiltrating lymphocytes (TILs), a marked reduction in TIGIT+Tregs populations, and ultimately, prolonged host survival (53). These findings further underscore the central role of TIGIT+Tregs in immune evasion and highlight the therapeutic potential of targeting both TIGIT and FAK pathways to enhance anti-tumor immunity. Nevertheless, the translational value of this combination strategy awaits validation in human studies, and the potential off-target toxicity of systemic FAK inhibition remains a concern. Whether FAK expression levels could serve as a predictive biomarker for TIGIT-targeted therapy also requires further investigation. Furthermore, TIGIT^+^Tregs exhibit a distinct gene expression profile compared to TIGIT^-^Tregs and demonstrate enhanced immunosuppressive activity. In contrast to their TIGIT- counterparts, TIGIT^+^Tregs possess a greater capacity to suppress immune responses, further reinforcing their role in immune evasion within the TME (54). Of note, this transcriptomic analysis was performed on Tregs from healthy donors rather than cancer patients, and whether the same expression signatures apply to tumor-infiltrating Tregs in ovarian cancer remains unknown. Additionally, it is unclear whether TIGIT^+^ Tregs represent a stable differentiation lineage or a dynamically inducible state, a distinction with important therapeutic implications.

Dendritic cells (DCs) are key regulators of the immune system, responsible for capturing and processing antigens and modulating immune responses through cytokine secretion. They play a crucial role in the activation of T cells and NK cells, thereby initiating adaptive immunity (55). However, in their immature state, DCs exhibit limited antigen-presenting capacity and tend to secrete immunosuppressive cytokines, such as interleukin-10 (IL-10) and transforming growth factor-beta (TGF-β), which contribute to immune tolerance and regulation (56). Interestingly, DCs highly express CD155 on their surface, a molecule that interacts with various ligands on T cells and NK cells, triggering distinct functional effects (57). Notably, when TIGIT binds to CD155, it induces tyrosine phosphorylation at the cytoplasmic tail of CD155, activating downstream signaling pathways and altering cytokine secretion in DCs. This signaling cascade shifts the cytokine profile from pro-inflammatory IL-12p40 to immunosuppressive IL-10, thereby promoting an immunosuppressive phenotype, dampening immune activation, and facilitating immune evasion within the TME (18). However, these mechanistic insights are derived primarily from studies in non-cancer settings or other tumor types, and direct evidence for the TIGIT-CD155 axis mediating DC-driven immunosuppression in ovarian cancer is currently lacking. Whether the TIGIT-CD155 axis similarly mediates DC-driven immunosuppression in ovarian cancer remains to be fully elucidated. Specifically, it is unknown whether CD155 expression on ovarian cancer-infiltrating DCs is upregulated or functionally distinct compared to DCs in other malignancies, and whether the TIGIT-induced IL-10 shift occurs with similar magnitude in the ovarian TME, which is characterized by unique cytokine and metabolic profiles. Future studies should further investigate the expression dynamics of CD155 on dendritic cells within the ovarian cancer tumor microenvironment and explore the molecular mechanisms underlying TIGIT-dependent immunosuppression. Gaining such insights may reveal novel targets and therapeutic strategies for tumor immunotherapy.

TIGIT can also alter the phenotype of macrophages. Research by Brauneck et al. has demonstrated that the frequency of TIGIT expression on macrophages in HGSOC is significantly higher compared to healthy donors. TIGIT blockade markedly reduced the frequency of M2-polarized macrophages. Furthermore, dual blockade of TIGIT and CD47 significantly enhanced the phagocytic activity of tumor-associated macrophages (TAMs) against ovarian cancer cells, outperforming single CD47 blockade (58). These findings suggest that targeting TIGIT not only modulates macrophage polarization but also synergizes with CD47 inhibition to promote anti-tumor immunity in HGSOC.

### 3.2 CD155-CD226

CD226 is a transmembrane glycoprotein widely expressed on various immune cells, including T lymphocytes and NK cells. As a critical immunoreceptor, it plays a pivotal role in cellular signaling and immune regulation. Clinical studies have demonstrated that its expression on CD8+ T lymphocytes is strongly associated with favorable prognostic outcomes (25).

CD226, as a multifunctional immune molecule, not only mediates the adhesion between immune cells and target cells and promotes the formation of immune synapses (59, 60), but also regulates cytotoxic responses and cytokine secretion through intracellular signal transduction mechanisms (61). Specifically, CD226 exerts its biological functions by binding to Grb2, activating the downstream ERK and AKT kinase pathways, and triggering calcium influx (61). However, in the TME, the function of CD226 is often limited by multiple factors. Firstly, TIGIT competitively binds to the same ligand CD155 as CD226, and TIGIT has a higher affinity for CD155. This competitive inhibition significantly weakens the activation effect of CD226 (62). Additionally, TIGIT can directly bind to CD226, inhibiting its dimerization process, which is a critical step for CD226 to function properly (12). Secondly, the downregulation or loss of CD155 expression in tumor cells prevents CD226 from functioning normally, constituting one of the important mechanisms of tumor immune escape (63).

In healthy tissues, NK cells undergo a “licensing” mechanism that enhances CD226 expression while reducing expression of inhibitory killer-cell immunoglobulin-like receptors (KIRs), creating an immunologically favorable CD226-to-inhibitory KIR ratio that establishes cellular activation potential. Within malignant solid tumors, this crucial immunoregulatory pathway becomes dysregulated - with compromised CD226 upregulation - ultimately permitting neoplastic escape from NK cell-mediated immune monitoring (64). Notably, research findings demonstrate that in endometrial cancer, artesunate induces upregulation of CD155 expression through autophagy-mediated mechanisms. This subsequently enhances the cytotoxicity of NK cell line NK92 via increased expression of the co-stimulatory molecule CD226 (44). It reveals the important role of the CD155/CD226 interaction in mediating the enhanced cytotoxicity of NK cells in endometrial cancer. However, these findings are derived from endometrial cancer and the NK92 cell line, and whether artesunate exerts similar effects on primary NK cells in ovarian cancer remains unknown. Additionally, the translational potential of artesunate as an immunomodulatory agent in ovarian cancer would require validation in patient-derived samples and *in vivo* models. Similarly, NK cells in the peritoneal fluid of epithelial ovarian cancer exhibit significantly reduced CD226 expression, a change closely associated with impaired cytotoxic function (65). TGF-β within the TME plays a key role in this suppression, further downregulating CD226 on NK cells and contributing to their functional exhaustion (66). Interestingly, this immunosuppressive effect of TGF-β is also reinforced by platelet involvement, as platelets have been shown to assist ovarian cancer cells in evading NK cell-mediated immune attack. Specifically, platelet-derived TGF-β inhibits CD226 expression on NK cells, thereby dampening their antitumor activity (67). Further research has demonstrated that TGF-β1 impairs NK cell function by promoting Smad2/3 phosphorylation, which in turn downregulates the expression of key activating receptors like CD226, as well as cytotoxic mediators. However, this suppression can be reversed by an IL-15 superagonist complexed with IL-15 receptor alpha (IL-15SA/IL-15RA), which inhibits Smad2/3-mediated transcriptional activity. This blockade effectively reinstates the expression of CD226 and other activating molecules, thereby restoring the cytotoxic capabilities of NK cells (68). Nevertheless, the therapeutic application of IL-15SA/IL-15RA in ovarian cancer requires careful evaluation. Systemic IL-15 stimulation may carry risks of cytokine release syndrome or unintended activation of immunosuppressive cell populations, such as regulatory T cells. Furthermore, the efficacy of this approach in the complex ovarian tumor microenvironment, where multiple suppressive factors coexist, has not been directly tested. In addition, it has been observed that tumor-derived exosomes contribute to the suppression of CD226 expression on infiltrating NK cells (69). Further studies are needed to explore whether ovarian cancer-derived exosomes exert a similar regulatory effect on CD226 levels in NK cells, and to identify the specific exosomal cargo involved in this process using techniques such as RNA sequencing or mass spectrometry. Notably, the exosome studies cited were not performed in ovarian cancer models, and the specific molecular cargo responsible for CD226 downregulation has not been identified in any gynecologic malignancy. Elucidating these mechanisms could inform the development of strategies to block exosome-mediated immune suppression in ovarian cancer.

**Figure 3:**
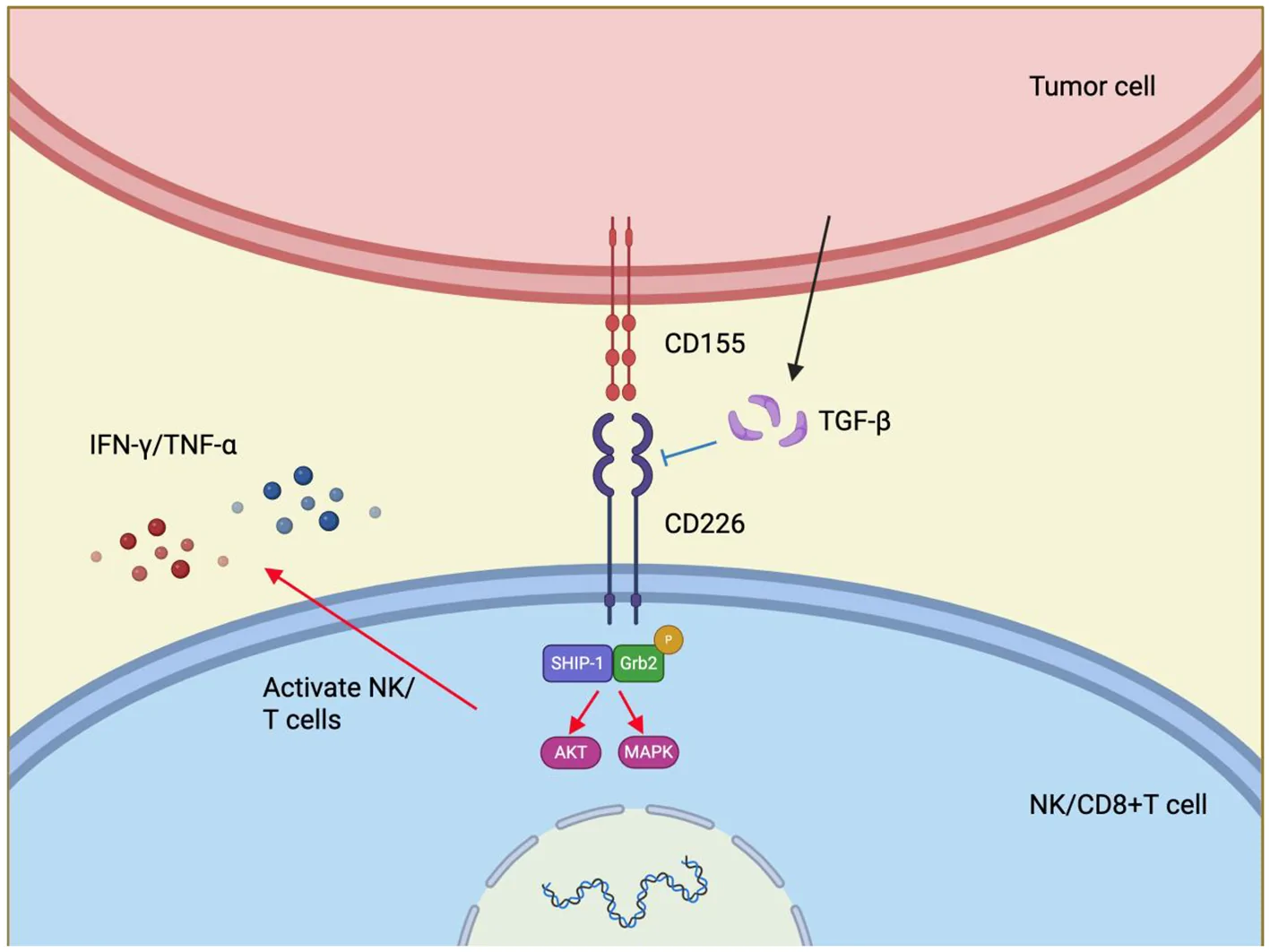
Interaction of CD155 with CD226 in CD8^+^T and NK cells. CD155 expressed on tumor cells engages the activating receptor CD226 on CD8^+^ T cells and NK cells. CD226 is a transmembrane glycoprotein containing two IgV domains arranged in parallel and an intracellular ITT-like signaling motif. Upon ligation, CD226 recruits the adaptor Grb2, activating downstream ERK and AKT kinase pathways and triggering calcium influx. This signaling cascade promotes immune synapse formation, enhances cytotoxic activity, and increases secretion of effector cytokines (IFN-γ, TNF-α). ERK, extracellular signal-regulated kinase; AKT, protein kinase B.

CD226 functions as a key activating receptor not only in NK cells but also in CD8+T cells. Evidence suggests that CD226 is critical for promoting CD8+T cell activation, particularly under conditions where DC antigen presentation is lacking. However, its role diminishes when DCs effectively present antigens (70), indicating that CD226 contributes to broadening the functional flexibility of CD8+T cells. In acute myeloid leukemia and similar contexts, studies have observed an increased proportion of CD8+T cells expressing the inhibitory receptor TIGIT, accompanied by a reduction in CD226 expression. This shift in surface receptor expression impairs the cytotoxic capabilities of CD8+T cells and suppresses the production of key effector cytokines such as IFN-γ and TNF-α, correlating with poor clinical outcomes (71). Moreover, some reports indicate that CD155 expressed on tumor cells may facilitate immune evasion by promoting the degradation of CD226 in CD8^+^T cells, thereby contributing to resistance against immunotherapeutic interventions (24). However, the evidence for CD155-mediated CD226 degradation in CD8^+^ T cells has been primarily demonstrated in melanoma models (24), and whether this mechanism operates in ovarian cancer remains unexplored. Additionally, the study by Braun et al. focused on the role of tumor cell-intrinsic CD155 in driving CD226 downregulation, but it did not fully address whether other microenvironmental factors, such as TGF-β or hypoxia, synergize with CD155 to promote CD226 degradation. It is also unclear whether the extent of CD226 loss on CD8^+^ T cells correlates with resistance to PD-1/PD-L1 inhibitors in ovarian cancer patients, as has been suggested in other tumor types. Furthermore, while the TIGIT/CD226 ratio on CD8^+^ T cells has been proposed as a prognostic marker in AML (71), whether this ratio holds predictive value in ovarian cancer has not been established, given the distinct immune landscapes and mutational profiles of these malignancies. A key focus of future research lies in examining the expression levels of CD226 on CD8+T cells within the ovarian cancer TME and determining whether tumor-expressed CD155 similarly mediates the degradation of CD226 on these cells (**Figure 3**).

### 3.3 CD155-CD96

The precise function of CD96 remains incompletely understood; however, two distinct structural motifs have been identified. One is the ITIM motif, which is involved in the transmission of inhibitory signals (72), and the other is the YXXM motif, which plays a role in receptor activation (28). CD96 expression is closely associated with immune infiltration and may serve as a co-stimulatory molecule in immunosuppression or immune adhesion (73). Similar to TIGIT, CD96 functions as both a risk factor and a protective factor in different tumor types (74). A study has shown that CD96, highly expressed in solid tumors, regulates Stat3 phosphorylation via its YXXM motif, thereby activating the Src-Stat3-Opa1 signaling pathway. This activation promotes mitochondrial membrane remodeling, enhances mitochondrial fatty acid β-oxidation, and ultimately contributes to increased chemoresistance in cancer stem cells (75). Recent studies have shown that the interaction between CD96 and its ligand CD155 can inhibit the antitumor activity of NK cells. However, the function of CD96 in human T cells is still a subject of debate (11, 76).

Moreover, in cervical cancer, elevated CD96 expression has been observed on CD8^+^TILs from patients who are unresponsive to PD-1 blockade. In PD-1 blockade-resistant tumors, surface levels of CD96 on CD8+TILs are significantly increased. Targeting CD96 in these settings markedly enhances the efficacy of PD-1 blockade, suppresses tumor growth, and restores the function of CD8^+^TILs in both murine models and human cervical cancer specimens. However, these findings are derived from cervical cancer, and whether CD96 upregulation serves as a resistance mechanism to PD-1 blockade in ovarian cancer remains unknown, given the substantial differences in immune microenvironment and mutational profiles between these malignancies. Is CD96 also associated with CD8^+^T cell exhaustion in other gynecologic cancers? This remains an important question that warrants further investigation in the future. Interestingly, a study in melanoma reported that blocking the interaction between CD96 and tumor-expressed CD155 impaired antigen-specific CD8^+^ T cell responses (29). It was further demonstrated that CD96 could deliver an activating signal through the MEK-ERK pathway, promoting the accumulation of NUR77^+^ and T-bet^+^CD8^+^T cells and enhancing their cytotoxic activity (29). This observation reveals a striking dichotomy, as CD96 appears to exert opposing functions in cervical cancer versus melanoma, suggesting that its role may be highly context-dependent and shaped by the specific TME. Whether a similar dual role of CD96 exists in gynecological cancers, and how it may be shaped by the tumor microenvironment, remains to be determined. This represents an important avenue for future investigation.

## 4 Treatment strategies for ovarian cancer focusing on CD155 and its receptors

Several strategies for antitumor therapies targeting CD155 and its receptors are currently under investigation (73, 77) (**Table 1**). These include the use of recombinant poliovirus to selectively target tumor cells that overexpress CD155, blocking inhibitory receptors of CD155 using monoclonal antibodies (mAb), and enhancing effector cell activation via CD226 through genetic modification or *in vitro* induction (CD226-based T-cell therapy) (74).

**Table 1:** Clinical trials targeting CD155 and its ligand.

Type/target	Combination therapy	Cancer type	Clinical trial	Phase
Virotherapy/CD155	PVS-RIPO	Recurrent Malignant Glioma	NCT02986178	2
Virotherapy/CD155	Atezolizumab (Anti–PD-L1)	Recurrent Malignant Glioma	NCT03793789	1b/2
Virotherapy/CD155	PVS-RIPO	Invasive Breast Cancer	NCT03564782	1
Virotherapy/CD155	PVS-RIPO	Melanoma	NCT03712358	1
Virotherapy/CD155	Nivolumab (Anti–PD-1)	Melanoma	NCT04125719	1
Virotherapy/CD155	PVS-RIPO	GBM	NCT01491893	1
Virotherapy/CD155	PVS-RIPO (Lerapolture), Anti–PD-1/L1	Solid Tumors	NCT04690699	1/2
Virotherapy/CD155	PVS-RIPO	Melanoma	NCT04577807	2
Virotherapy/CD155	PVS-RIPO (Lerapolture), Anti–PD-1	GBM (Recurrent and Supratentorial)	NCT04479241	1
Virotherapy/CD155	PVS-RIPO	Recurrent Malignant Glioma in Children	NCT03043391	1
Monoclonal antibodies/CD155	NTX-1088 + Pembrolizumab (Anti–PD-1)	Solid Tumors	NCT05378425	1
Monoclonal antibodies/CD155	COM701, BMS-986207	Advanced Solid Tumors	NCT04570839	1/2
Monoclonal antibodies/CD155	COM701 + Nivolumab (Anti–PD-1)	Advanced Solid Tumors	NCT03667716	1
Monoclonal antibodies/TIGIT	TAB006 + Toripalimab (Anti–PD-1)	Advanced Solid Tumors/advanced lymphoma	NCT05253105	1
Monoclonal antibodies/TIGIT	PM1021, PM8001	Advanced Solid Tumors	NCT05537051	1
Monoclonal antibodies/TIGIT	JS006 + Toripalimab (Anti–PD-1)	Advanced Tumors	NCT05061628	1
Monoclonal antibodies/TIGIT	BMS-986207, BMS-986016, Elotuzumab (Anti–SLAMF7)	Multiple Myeloma	NCT04150965	1/2
Monoclonal antibodies/TIGIT	Tiragolumab + Atezolizumab (Anti–PD-L1)	High-Risk Urothelial Carcinoma	NCT05394337	1
Monoclonal antibodies/TIGIT	Ociperlimab + Atezolizumab (Anti–PD-L1)	NSCLC	NCT04746924	3
Monoclonal antibodies/TIGIT	Tislelizumab + Atezolizumab (Anti–PD-L1)	NSCLC	NCT04294810	3
Monoclonal antibodies/TIGIT	Tiragolumab + Atezolizumab (Anti–PD-L1)	Esophageal Squamous Cell Carcinoma	NCT04543617	3
Monoclonal antibodies/TIGIT	Tiragolumab + Atezolizumab (Anti–PD-L1) + Chemotherapy	SCLC	NCT04256421	3
Monoclonal antibodies/TIGIT	Domvanalimab + Zimberelimab (Anti–PD-1)	Advanced Upper Gastrointestinal	NCT05568095	3
Monoclonal antibodies/TIGIT	Nivolumab (Anti–PD-1) + Chemotherapy	Lung Cancer	NCT04736173	3
Monoclonal antibodies/TIGIT	Tislelizumab + Ociperlimab	NSCLC	NCT04866017	3

### 4.1 Monoclonal antibodies related to CD155

The involvement of CD155 is crucial in the development and infiltration of tumors, making its blockade an effective therapeutic approach (78). Ongoing research is investigating the potential of CD155 as a viable target for cancer immunotherapy. For instance, Zhao et al. examined the efficacy of a novel bispecific antibody directed against both CD3 and CD155 (CD155 Bi-Ab) in enhancing the cytotoxic activity of activated T cells (ATCs) towards tumor cells. Their study showed that CD155 Bi-Ab significantly boosted the tumor-killing ability of ATCs. In particular, treatment with CD155 Bi-Ab led to increased secretion of IFN-γ, TNF-α, and IL-2, as well as elevated expression of the activation marker CD69 in ATCs (79). Interestingly, a recent study has also evaluated the specific cytotoxic activity of T cells armed with CD155 Bi-Ab against ovarian cancer cells. The results showed that CD155Bi-T cells exhibited significant cytotoxicity toward CD155-positive ovarian cancer cells. Specifically, the cytotoxicity of CD155Bi-T cells was 2.67 times higher than that of control T cells, indicating a marked enhancement in tumor-killing activity (80). While these results are encouraging, the studies were conducted *in vitro*, and whether CD155 Bi-Ab-armed T cells can maintain their enhanced cytotoxicity and persist within the immunosuppressive ovarian tumor microenvironment *in vivo* remains to be demonstrated. Additionally, Rediocide-A, a natural compound derived from traditional Chinese medicine, has been shown to overcome NK cell-mediated immune resistance in non-small cell lung cancer by downregulating CD155 expression (85), suggesting its potential for targeting CD155 in ovarian cancer as well. However, direct evidence for Rediocide-A efficacy in ovarian cancer is currently lacking, and its mechanism of action in the context of ovarian cancer requires validation. Moreover, CD155 inhibition has been found to restore DC function (57), offering a promising avenue for treating tumors resistant to conventional immune checkpoint inhibitors (ICIs) (57). However, a potential drawback of CD155 blockade is its disruption of the CD155-CD226 interaction, which may compromise therapeutic efficacy. To address this, clinical trials are investigating CD155-targeted therapies, including NTX-1088, the first anti-CD155 monoclonal antibody developed for solid tumors, which entered phase I trials in early 2022 (NCT05378425). NTX-1088 binds CD155 with high affinity, blocking its interactions with TIGIT and CD96 to inhibit their suppressive signaling. Additionally, it prevents CD155 from binding to CD226, thereby avoiding CD226 internalization, restoring its surface expression on immune cells, and enhancing antitumor immune responses. While the mechanistic design of NTX-1088 is conceptually appealing, its clinical development remains in early phase I, and ovarian cancer-specific expansion cohorts have not yet been reported. Similarly, the MAIA-ovarian trial evaluating the anti-PVRIG antibody COM701 as maintenance therapy in platinum-sensitive ovarian cancer is ongoing, with interim data anticipated in 2027; however, whether single-agent targeting of a secondary checkpoint pathway can meaningfully improve outcomes in this setting remains unproven. Preliminary results from a phase I cohort of platinum-resistant ovarian cancer patients receiving triple blockade of PVRIG, TIGIT, and PD-1 showed modest efficacy but were limited by small sample size and lack of a comparator arm, while a grade 3 immune-related adverse event highlighted potential safety concerns with such combinations.

Antibody-drug conjugates (ADCs) are an innovative therapeutic approach that combines monoclonal antibodies with cytotoxic agents. The FDA-approved brentuximab vedotin and trastuzumab have demonstrated the success of this approach in oncology. With advancements in ADC technology, over 50 ADCs are currently in clinical trials (81, 82). Developing ADCs targeting CD155 presents a promising strategy, especially given the encouraging responses observed in patients who have progressed after platinum-based chemotherapy or ICIs (83).

### 4.2 Targeting the receptors of CD155

CD155 interacts with several receptors, with TIGIT showing the highest affinity. This interaction forms a homodimer of CD155, which then pairs with TIGIT to form a heterotetramer. Blocking the TIGIT-CD155 binding can activate both T cells and NK cells, making this strategy a promising approach for cancer immunotherapy, particularly when combined with other ICIs. TIGIT has garnered significant attention due to its strong binding to CD155 and its overexpression in NK/T cells, which has accelerated NK cell-based therapies (84). The high affinity between TIGIT and CD155 interferes with the CD226-CD155 interaction, leading to NK cell inhibition (23). TIGIT’s role in immune suppression has been extensively studied, highlighting its potential as a target for immunotherapy (85). TIGIT binding to CD155 or CD112 inhibits NK cell cytotoxicity by reducing IFN-γ secretion and altering granule polarization, which affects the release of cytolytic granules (86). Blocking TIGIT has been shown to enhance NK cell function and improve immunotherapy outcomes.

NK cell therapy has shown promise for treating gynecological cancers (87, 88). Combining NK cell therapy with other treatments, including donor-derived NK cells and checkpoint inhibitors, is being tested in preclinical trials to counteract tumor-induced immunosuppression and improve NK cell responses in gynecological cancers (89). Both unmodified and engineered NK cells have been used in clinical trials targeting ligands overexpressed on tumor cells (85). Although no trials currently focus on targeting CD155 directly with NK or T cells, other tumor cell ligands have been extensively studied (90). Of note, these NK cell therapies primarily target other tumor-associated antigens rather than CD155, and evidence for their efficacy through the CD155 axis in ovarian cancer remains absent.

CD155 expression across various tumor types, along with the expression of TIGIT/CD96 and CD226 in infiltrating lymphocytes, can help guide the selection of cancer types for clinical trials. Targeting the inhibitory pathways of CD155, particularly through TIGIT and CD96, has shown favorable outcomes without the challenges of blocking CD155-CD226 interactions (32). TIGIT monoclonal antibodies have been shown to improve tumor cell clearance by lymphocytes (91). Similarly, targeting CD96 with CD8+ T and NK cells has been effective in experimental metastasis models, including glioma and melanoma (92). However, the exact role of CD96 and TIGIT in tumor growth inhibition remains uncertain, particularly regarding their redundancy (93). This redundancy is particularly relevant in ovarian cancer, as TIGIT and CD96 may exert differential dominant functions across distinct immune cell subsets or at different disease stages. Further mechanistic studies on CD155, CD226, CD96, and TIGIT are needed to better understand their combined impact on antitumor immune responses. Additionally, the overexpression of TIGIT and CD96 in tumor-infiltrating T cells has been linked to their terminal differentiation, further supporting their blockade for therapeutic purposes (92, 93). However, whether terminally differentiated T cells can be effectively reversed by blocking these receptors, or whether they have already entered an irreversible state of functional exhaustion, remains unclear—a distinction with important implications for therapeutic timing.

CAR-T cell therapies, particularly those using chimeric antigen receptors (CARs), have proven highly effective in cancer treatment (94). CAR-T cells are genetically modified to target tumor antigens, enabling them to bypass immune evasion mechanisms (95, 96). Fourth-generation CAR-T cells, which include additional cytokine production capabilities, have enhanced anti-tumor effects. Studies have shown that CD226-based CAR-T cells exhibit significant cytotoxicity against various tumor types both *in vitro* and *in vivo* (97). However, CAR-T cell therapy can trigger cytokine release syndrome (CRS), with severity dependent on factors such as the number of cells and specific CAR-T mechanisms (98). Despite this, CD226 CAR-T cells have shown promise in preclinical models, although further refinement is needed for clinical use (99). Beyond CRS, the clinical translation of CD226-based CAR-T cells faces additional challenges: heterogeneous CD155 expression across ovarian cancer lesions may contribute to antigen escape; the TGF-β-rich ascites and highly immunosuppressive tumor microenvironment may limit the persistence and function of adoptively transferred CAR-T cells; and the risk of on-target off-tumor toxicity, given CD155 expression on normal tissues, requires thorough evaluation in ovarian cancer-specific preclinical models. Addressing these challenges will be critical for determining whether CD155-axis-targeted cell therapies can translate into meaningful clinical benefits for ovarian cancer patients.

In conclusion, targeting the TIGIT/CD155 axis through cell engineering or monoclonal antibodies holds significant promise for enhancing NK cell-mediated anti-tumor responses. Combining TIGIT/CD155 inhibition with other immune checkpoint strategies may lead to more effective and durable clinical results. However, due to the complexity of the TME, simultaneous targeting of multiple immune checkpoints and cytokines might be necessary for optimal therapeutic efficacy. Nevertheless, the optimal combination regimen for such multi-targeted approaches has not been defined, and their safety profile, particularly in ovarian cancer patients, requires systematic evaluation in larger-scale trials.

## 5 Conclusion and future perspectives

CD155 has emerged as a critical immune checkpoint in ovarian cancer, functioning at the crossroads of immune regulation and tumor progression. By interacting with TIGIT and CD96 while competing with CD226, CD155 orchestrates a complex signaling network that drives T cell exhaustion, NK cell dysfunction, and regulatory T cell expansion, ultimately promoting immune evasion. Its expression on tumor cells, dendritic cells, and macrophages, as well as the presence of soluble isoforms, further contributes to the establishment of an immunosuppressive tumor microenvironment.

Therapeutic strategies aimed at disrupting the CD155 axis—whether by blocking inhibitory interactions or restoring CD226-mediated co-stimulation—are under active investigation. Monoclonal antibodies, bispecific constructs, and engineered CAR-T or NK cell therapies have demonstrated encouraging preclinical results and early-phase clinical potential. However, several challenges must be addressed before these approaches can be widely translated into clinical practice.

First, the dual role of CD155 as both an inhibitory and activating ligand complicates the design of targeted interventions. Non-selective blockade may unintentionally suppress beneficial CD226 signaling. Second, the regulation of CD155 expression is dynamic and influenced by tumor metabolism, cytokine signaling (e.g., TGF-β), and autophagy, raising the need to develop context-dependent therapeutic strategies. Third, biomarkers to predict patient response to CD155-targeted therapy remain lacking. Emerging evidence suggests that tumor CD155 expression, the TIGIT/CD226 ratio on tumor-infiltrating lymphocytes, and TIGIT^+^ Tregs frequency may serve as potential candidates, but these require prospective validation in ovarian cancer cohorts. It remains unclear whether a single marker or a composite panel would offer superior predictive value, and the dynamic nature of CD155 expression suggests that serial monitoring during treatment may be necessary. Additionally, soluble CD155 in plasma or ascites represents a potential non-invasive biomarker, though its clinical utility in ovarian cancer has not been established. Integrating these candidates into prospective trial designs will be essential for identifying patients most likely to benefit from CD155-targeted therapies. Moreover, the immunological effects of soluble CD155 and tumor-derived exosomes that modulate distant immune responses are still poorly understood. Whether these components contribute to resistance to checkpoint inhibitors or influence systemic immunity requires further investigation.

Looking forward, we envision several priority directions for future research. At the mechanistic level, the precise interplay between TIGIT, CD96, and CD226 in different immune cell subsets within the ovarian TME requires further elucidation, particularly regarding the relative contribution of each receptor under varying tumor microenvironmental conditions. At the translational level, biomarker-driven clinical trials incorporating baseline and on-treatment immune monitoring will be critical to validate predictive signatures and to understand resistance mechanisms. Notably, the MAIA-ovarian trial and ongoing studies of NTX-1088 and COM701 will provide important early clinical data that may inform subsequent trial design. At the therapeutic level, rational combination strategies integrating CD155-axis blockade with PD-1/PD-L1 inhibitors, chemotherapy, or FAK inhibitors may offer synergistic benefits by simultaneously targeting multiple layers of immune suppression. The emerging evidence linking autophagy and metabolic pathways to CD155 regulation also suggests that combining CD155-targeted agents with metabolic modulators could represent a novel therapeutic avenue. Finally, efforts to develop and validate non-invasive biomarkers, including soluble CD155 and exosomal cargo, are warranted, as they could facilitate patient stratification and enable dynamic monitoring of treatment response. Addressing these questions through rigorous translational research will be essential to determine whether the CD155 axis represents a transformative therapeutic opportunity in ovarian cancer.
